# Cable Defect Detection Using an Improved RT-DETR Model

**DOI:** 10.3390/s26196006

**Published:** 2026-09-22

**Authors:** Ziwei Yi, Ying Fu, Hua Xiang, Tongxi Wang

**Affiliations:** 1 School of Computer Science, Yangtze University, Jingzhou 434023, China; 2 Research Center for Digital Agriculture and Intelligent Engineering, Yangtze University, Jingzhou 434023, China; 3 Artificial Intelligence Research Platform, Yangtze University, Jingzhou 434023, China

**Keywords:** cable defect detection, object detection, RT-DETR, lightweight model, deep learning

## Abstract

Small defect targets and complex backgrounds in transmission-line inspection images can limit detection accuracy, and existing detection models often incur substantial computational cost. To address these issues, this study proposes a cable defect detection method based on an improved RT-DETR. First, a DualConv module is introduced into the backbone to strengthen local feature representation while reducing parameter redundancy. Second, a ContextGuidedDown module is incorporated for downsampling to alleviate the loss of critical information. Finally, Dynamic Tanh replaces LayerNorm in the attention-based intra-scale feature interaction (AIFI) encoder to improve the regulation of high-level features. Experiments were conducted on the CableInspect-AD dataset and the RF100 Cable Damage dataset. In the main single-run comparison on CableInspect-AD, the proposed method achieves an mAP@0.5 of 92.4%, which is 2.7 percentage points higher than that of the original RT-DETR, while the number of parameters is reduced from 20.1 M to 18.6 M and the computational cost decreases from 58.3 to 53.4 GFLOPs. Across four paired random-seed runs, DCGD-RTDETR achieves a mean mAP@0.5 of 92.05 ± 0.34%, compared with 90.98 ± 1.10% for RT-DETR; however, the paired difference is not statistically significant (*p* = 0.086). In the same repeated-run analysis, AP@0.75 decreases from 65.01 ± 1.43% for RT-DETR to 63.26 ± 1.11% for DCGD-RTDETR (*p* = 0.005), indicating that localization performance at a stricter IoU threshold remains a limitation. Ablation experiments further confirm the complementary contributions of the three modifications. Overall, the proposed method provides a favorable detection-accuracy and model-complexity trade-off at IoU = 0.5, while further improvement is needed for stricter-IoU localization performance.

## 1. Introduction

With the continued expansion of power systems, transmission-line cables serve as important carriers of electrical energy, and their operating condition directly affects the safety and stability of the power system [1]. Long-term exposure to rain, salt-fog corrosion, temperature variation, and mechanical vibration can cause surface defects and structural anomalies, including breaks, wear, scratches, deformation, and foreign-material deposits. These defects may compromise structural integrity, reduce current-carrying capacity, create local stress concentrations, and degrade insulation performance. If they are not detected and addressed promptly under complex operating conditions, they may lead to partial discharge, equipment failure, or even large-scale power outages. Reliable cable defect detection is therefore of considerable engineering importance.

Conventional cable defect detection mainly relies on manual inspection, electromagnetic testing, and infrared inspection [2,3,4,5]. These methods often depend on operator experience and specialized equipment, resulting in low efficiency, strong subjectivity, and relatively high miss rates. They can also be difficult to apply efficiently in complex field inspection environments and may be affected by illumination variation and background interference. In addition, conventional methods have limited capability in identifying very small defects and detecting multiple defect categories simultaneously, making them insufficient for modern intelligent transmission-line inspection.

### Related Work and Research Gap

With the rapid development of computer vision and deep learning, object detection has become an important approach for automated inspection of transmission lines and cable-related components [6,7,8,9,10]. Recent studies have increasingly focused on improving detection accuracy against complex backgrounds and for small or subtle defects. Yang et al. [11] developed an improved YOLOv9-based detector for high-voltage transmission-line defect detection, incorporating image enhancement and attention mechanisms to improve small-target recognition under challenging visual conditions. Notably, Yang et al. [11] also incorporated DualConv into an improved YOLOv9 framework, together with image enhancement and attention mechanisms, for cable-defect inspection. Therefore, the use of DualConv itself is not claimed as a novel contribution of this study; the contribution here lies in its task-oriented integration with ContextGuidedDown and DyT within RT-DETR and in the systematic evaluation of their complementary effects. Liu et al. [12] proposed an improved YOLOv10 method for steel-cable surface defect detection, using attention and multi-feature fusion to reduce missed and false detections caused by background similarity. Wei et al. [13] combined CNN-based feature extraction with Transformer-based cross-scale feature fusion for high-voltage cable surface defect detection, demonstrating the potential of hybrid architectures for fine-grained defect representation. These studies confirm the effectiveness of deep-learning-based detectors for cable-related inspection, but challenges remain in simultaneously handling subtle defect morphology and complex backgrounds while maintaining computational efficiency. Beyond cable-related inspection, related object-detection studies have also explored attention and uncertainty-aware learning under challenging conditions. Yuan et al. [14] enhanced AVOD with channel, spatial, and mixed attention for 3D vehicle detection, while Luo et al. [15] used uncertainty-guided collaborative learning for nighttime object detection. These studies provide broader context for feature enhancement under difficult visual conditions.

In parallel, several general-purpose detection and feature-processing techniques provide useful mechanisms for improving this accuracy–complexity trade-off. RT-DETR [16] provides a real-time end-to-end detection framework and is adopted as the baseline in this study. DualConv [17] uses parallel grouped and pointwise convolutions to improve lightweight feature extraction, while context-guided feature modeling [18] combines local and surrounding contextual information. Dynamic Tanh (DyT) [19] provides an alternative feature-regulation mechanism for Transformer architectures without explicitly computing feature means and variances. These methods were originally developed for broader vision tasks rather than specifically for the cable-defect setting considered here.

Based on these observations, the research gap addressed in this study is not the invention of the above individual modules, but their task-oriented integration into RT-DETR for multi-category cable defect detection. Specifically, DualConv is introduced into the backbone for lightweight low-level feature extraction, ContextGuidedDown is used in the downsampling path to strengthen contextual representation, and DyT replaces LayerNorm in AIFI for high-level feature regulation. The resulting DCGD-RTDETR is systematically evaluated through component comparisons, complete combination ablation, multi-seed robustness analysis, strict-IoU metrics, computational-complexity assessment, and external validation. This design aims to investigate whether complementary modifications at different stages of RT-DETR can provide a favorable accuracy–complexity trade-off for cable defect detection.

## 2. Method

### 2.1. Overall Architecture

RT-DETR [16] is used as the baseline framework. To address insufficient feature representation, information loss during downsampling, and limited regulation of feature distributions in cable defect detection, the model is modified in three aspects: feature extraction, feature fusion, and feature regulation. The overall architecture of DCGD-RTDETR is shown in Figure 1.

DualConv is applied in the backbone feature-extraction stage, ContextGuidedDown is used for downsampling and feature fusion in the neck, and DyT regulates features in the AIFI module by replacing LayerNorm in the Transformer encoder. The three modules operate at different stages and are functionally complementary. In the backbone, each original BasicBlock is replaced by a BasicBlock_DualConv to improve low-level feature representation. In the neck, the downsampling convolutions in the path aggregation network (PAN) are replaced by ContextGuidedDown modules to preserve critical details and strengthen contextual modeling while reducing spatial resolution. At the high-level feature-regulation stage, the original AIFI module is replaced by AIFI-DyT, in which the two LayerNorm layers of the Transformer encoder are replaced by Dynamic Tanh. Together, these modifications improve cable-defect representation while maintaining a relatively low computational cost.

### 2.2. DualConv Module

In the original RT-DETR, the backbone mainly uses standard convolutional blocks for feature extraction. To improve the balance between representational capacity and computational efficiency, DualConv [17] is introduced into the backbone to modify the original BasicBlock. DualConv is a lightweight two-branch convolutional structure that applies a 3 × 3 grouped convolution and a 1 × 1 pointwise convolution in parallel to the same input feature map.

Let the input feature map be *X* with size *B* × *C*_in_ × *H* × *W*. The grouped-convolution branch extracts local spatial features as

(1) $$F_{g}={\mathrm{Conv}}_{3\times 3}^{\left(g\right)}(X;W_{g})$$

where *g* is the number of groups and *W*_g_ denotes the parameters of the grouped convolution. The pointwise branch performs cross-channel interaction and feature reorganization as

(2) $$F_{p}={\mathrm{Conv}}_{1\times 1}(X;W_{p})$$

where *W*_p_ denotes the parameters of the pointwise convolution. The outputs of the two branches have the same spatial and channel dimensions and are fused by element-wise addition:

(3) $$Y=\mathrm{DualConv}(X)=F_{g}+F_{p}$$

The parallel structure therefore combines local spatial feature extraction with cross-channel information interaction at relatively low computational cost. In the proposed detector, DualConv is used in the backbone BasicBlock to strengthen low-level cable-defect representation while reducing convolutional redundancy. The DualConv structure is shown in Figure 2.

### 2.3. ContextGuidedDown Module

In the original RT-DETR, the path aggregation network (PAN) in the neck uses a standard 3 × 3 convolution with stride 2 for downsampling. Although this operation is computationally efficient, it can discard fine details when reducing spatial resolution and provides limited explicit contextual modeling. To alleviate this problem, a ContextGuidedDown module based on context-guided feature modeling [18] is incorporated into the RT-DETR neck to replace the standard downsampling convolutions in the PAN path.

Given an input feature map X with size B × C × H × W, the module first applies a 3 × 3 convolution with stride 2 to reduce the spatial resolution and expand the channel dimension, producing

(4) $$F_{0}={\mathrm{Conv}}_{3\times 3,s=2}(X)$$

Two parallel depthwise-convolution branches are then constructed from F0. The local branch extracts neighborhood information using a standard 3 × 3 depthwise convolution:

(5) $$F_{\mathrm{loc}}={\mathrm{DWConv}}_{3\times 3}(F_{0})$$

The surrounding-context branch uses a dilated 3 × 3 depthwise convolution with dilation rate r to enlarge the effective receptive field:

(6) $$F_{\mathrm{sur}}={\mathrm{DWConv}}_{3\times 3,d=r}(F_{0})$$

The two branch features are concatenated along the channel dimension, followed by batch normalization, nonlinear activation, and a 1 × 1 convolution for feature fusion and channel adjustment:

(7) $$F_{\mathrm{joint}}={\mathrm{Conv}}_{1\times 1}\left(\delta \left(\mathrm{BN}\left(\left[F_{\mathrm{loc}},F_{\mathrm{sur}}\right]\right)\right)\right)$$

where [·, ·] denotes channel concatenation, BN(·) denotes batch normalization, and δ(·) denotes the nonlinear activation function. A global feature-recalibration operation is subsequently applied:

(8) $$Y=F_{\mathrm{Glo}}(F_{\mathrm{joint}})$$

where FGlo(·) denotes the global feature-recalibration operation implemented in the context-guided block. This compact formulation represents the implemented global recalibration stage without unnecessarily expanding its internal sub-operations. By combining local details, surrounding context, and global feature recalibration, ContextGuidedDown improves contextual representation during downsampling while retaining the original downsampling role of the PAN path. The ContextGuidedDown structure is shown in Figure 3.

### 2.4. Dynamic Tanh Module

In the original RT-DETR, the AIFI module uses a Transformer encoder for high-level feature modeling. Multi-head self-attention establishes interactions among spatial positions, the feed-forward network performs further feature transformation, and two LayerNorm operations regulate the outputs of the attention and feed-forward sublayers. To modify this feature-regulation mechanism, Dynamic Tanh (DyT) [19] is introduced to replace the two LayerNorm layers, forming the AIFI-DyT module. The remaining Transformer structure, including positional encoding, multi-head self-attention, feed-forward processing, and residual connections, is retained.

For an input feature x, DyT is defined as

(9) DyT(x)=γ⊙tanh(αx)+β

where α is a learnable scalar controlling the input scale before the hyperbolic tangent function, and γ and β are learnable channel-wise scale and bias parameters. The symbol ⊙ denotes element-wise multiplication. Unlike LayerNorm, DyT does not compute feature means or variances during the forward pass; instead, it applies a learnable nonlinear transformation followed by channel-wise scaling and shifting.

In AIFI, the two-dimensional feature map is reshaped into a token sequence of size B × N × C, where N = H × W. Positional encoding is retained exactly as in the original AIFI implementation and is omitted from the following equations for brevity. Replacing the two LayerNorm operations with DyT gives

(10) $$X_{1}={\mathrm{DyT}}_{1}\left(X+\mathrm{MHSA}(X)\right)$$

(11) $$Y={\mathrm{DyT}}_{2}\left(X_{1}+\mathrm{FFN}(X_{1})\right)$$

where MHSA denotes multi-head self-attention and FFN denotes the feed-forward network. The two residual connections remain unchanged. DyT therefore does not participate in attention-weight computation and does not introduce additional interactions among feature positions; global information exchange continues to be performed by the original multi-head self-attention. The AIFI-DyT structure is shown in Figure 4.

## 3. Datasets

### 3.1. CableInspect-AD Dataset

CableInspect-AD [20] is a visual anomaly detection dataset for power transmission-line inspection. It was constructed using real cables used in the field imaged in a controlled apparatus designed to simulate robotic inspection, with controlled background and illumination conditions. The dataset contains several types of defects that can occur during long-term cable operation.

The dataset covers multiple cable types and visual characteristics under operating conditions, including complex textures and background interference, which make the detection task challenging. It contains seven cable defect categories, as shown in Figure 5: broken strand, welded strand, bent strand, long scratch, crushed, spaced strand, and deposit. These categories differ substantially in morphology and scale and include fine structural changes, continuous damage, and regional anomalies.

The original CableInspect-AD dataset contains 4798 images, including 2639 anomalous images and 2159 normal images. The original CableInspect-AD publication reports 6023 annotations [20]. For the object-detection experiments in this study, we directly counted the bounding-box annotations contained in the COCO-format training, validation, and test files, obtaining 2685, 786, and 393 bounding boxes, respectively, for a total of 3864 defect instances across the seven categories. Thus, the value of 6023 reported for the original dataset and the 3864 bounding-box instances used in our experiments correspond to different annotation statistics and should not be interpreted as directly equivalent. No additional manual exclusion of defect categories or bounding boxes was performed after constructing the COCO-format detection splits; all bounding-box annotations present in these files were included. The dataset was divided into training, validation, and test sets containing 3356, 961, and 481 images, respectively, corresponding to 69.95%, 20.03%, and 10.03% of the data. The subsets were constructed to maintain comparable data distributions for fair evaluation.

The dataset exhibits class imbalance. Spaced strand is the most frequent category, with 1309 instances, whereas bent strand and welded strand contain only 163 and 288 instances, respectively, as shown in Figure 6. This imbalance increases the difficulty of learning, particularly for underrepresented categories. Across all images, the dataset contains approximately 0.81 defect instances per image, indicating that defect targets are relatively sparse and increasing the difficulty of localization and recognition in complex backgrounds.

Overall, CableInspect-AD combines diverse defect categories, imbalanced class distributions, and sparse targets. These characteristics place substantial demands on feature representation, multiscale modeling, and robustness, making the dataset suitable for evaluating the proposed cable defect detector.

### 3.2. RF100 Cable Damage Dataset for External Validation

To assess the applicability of the proposed model under a different data distribution, the RF100 Cable Damage dataset [21] is used for external validation. This object detection dataset focuses on cable damage and differs from CableInspect-AD in category definitions, image characteristics, and sample distribution, making it suitable for complementary evaluation.

The RF100 Cable Damage subset is a community-contributed object-detection dataset hosted on Roboflow Universe and incorporated into the RF100 benchmark. The public dataset metadata identify the original project creator and dataset composition, but do not provide detailed information on the physical image-acquisition setup; therefore, no specific camera or acquisition conditions are assumed in this study.

The dataset used in this study contains two annotated defect categories, namely break and thunderbolt. According to the label statistics, the dataset contains 1512 annotated defect instances in total, including 822 break instances and 690 thunderbolt instances. Specifically, the training set contains 577 break and 480 thunderbolt instances, the validation set contains 159 break and 149 thunderbolt instances, and the test set contains 86 break and 61 thunderbolt instances. Compared with CableInspect-AD, this dataset has fewer defect categories but a different data distribution, providing a complementary setting for evaluating model behavior outside the primary CableInspect-AD experiments.

The dataset is organized into training, validation, and test sets for model training, parameter selection, and final performance evaluation. The same experimental protocol used for CableInspect-AD is applied, and model performance is evaluated using mAP@0.5, mAP@0.5:0.95, the number of parameters, and FLOPs.

Experiments on the RF100 Cable Damage dataset therefore provide a complementary assessment of DCGD-RTDETR under a different cable-damage data distribution and allow its accuracy–complexity behavior to be examined beyond the primary CableInspect-AD experiments.

## 4. Experimental Setup and Evaluation Metrics

### 4.1. Experimental Environment

Experiments were conducted on a server equipped with two Intel Xeon Gold 6330 processors operating at 2.00 GHz and an NVIDIA GeForce RTX 3090 GPU with approximately 24 GB of memory. The operating system was Linux 5.15.0–94-generic. Models were developed and trained using Python 3.10 and PyTorch 2.2.2, with CUDA 12.1 and cuDNN 8.9.2 for GPU acceleration. The experimental environment is summarized in Table 1.

### 4.2. Training Protocol and Comparison Fairness

For the RT-DETR baseline and all RT-DETR-based variants, including the ablation models and DCGD-RTDETR, the same training protocol was used unless otherwise stated. Training was performed with images at an input size of 640, with a batch size of 16 for 250 epochs. AdamW was used with an initial learning rate of 1.0 × 10−4, a final learning-rate factor of 1.0, a momentum setting of 0.9, and a weight decay of 1.0 × 10−4. Four data-loader workers were used, pretrained initialization was enabled consistently for the RT-DETR family, and automatic mixed precision was disabled. The main single-run experiments used random seed 0. For the robustness experiment, the RT-DETR baseline and DCGD-RTDETR were independently retrained using seeds 1, 2, 3, and 4 while all other settings were kept unchanged. Validation data were used for model selection, and the final reported performance was obtained on the held-out test set.

For the representative-detector comparison, all models used the same CableInspect-AD training, validation, and test partitions (3356/961/481 images), an input size of 640, and the same held-out test-set evaluation protocol. Architecture-specific optimization settings were retained rather than forcing identical hyperparameters across fundamentally different detector families. YOLOv8n and YOLOv11n used the same training configuration: 300 epochs, batch size 32, SGD with an initial learning rate of 0.01, a final learning-rate factor of 0.01, momentum 0.937, weight decay 0.0005, pretrained initialization, automatic mixed precision enabled, and random seed 0. Faster R-CNN used a ResNet-50-FPN backbone with COCO-pretrained weights, batch size 4, SGD with a learning rate of 0.005, momentum 0.9, weight decay 0.0005, and random seed 0. Validation-based early stopping was used for Faster R-CNN, and the checkpoint with the best validation performance was obtained at epoch 25 and evaluated on the same test set. The principal configurations are summarized in Table 2.

The CableInspect-AD data used in this study are class-imbalanced. No explicit class re-weighting, oversampling, undersampling, or class-aware sampling strategy was introduced. The RT-DETR optimization framework uses Varifocal Loss for classification together with a focal-style classification cost during Hungarian matching; these mechanisms are components of the original detector optimization rather than additional class-balancing strategies introduced in this study. Per-class detection results are therefore reported throughout the experiments to make the effect of the uneven class distribution transparent.

### 4.3. Evaluation Metrics

Detection performance is evaluated using complementary accuracy metrics rather than relying on a single IoU threshold. Throughout this paper, AP@0.5 denotes class-wise average precision at IoU = 0.5, whereas mAP@0.5 denotes the mean of AP@0.5 over all defect categories. Following common COCO-style notation, AP@0.75 denotes the category-averaged AP at IoU = 0.75, and mAP@0.5:0.95 summarizes performance averaged over IoU thresholds from 0.50 to 0.95. For N classes, mean average precision is defined as

(12) $$\mathrm{mAP}=\frac{1}{N}\sum_{i=1}^{N}{\mathrm{AP}}_{i}$$

where APi denotes the average precision of class i. In continuous form, AP corresponds to the area under the precision-recall curve:

(13) $${\mathrm{AP}}_{i}=\int_{0}^{1}p_{i}(r)dr$$

where pi(r) denotes precision as a function of recall for class i. Precision and recall are defined as

(14) $$\mathrm{Precision}=\frac{TP}{TP+FP},\;\;\mathrm{Recall}=\frac{TP}{TP+FN}$$

where TP, FP, and FN denote true positives, false positives, and false negatives, respectively. AP@0.75 is computed at an IoU threshold of 0.75. The COCO-style mAP@0.5:0.95 metric averages mAP over ten IoU thresholds from 0.50 to 0.95 in increments of 0.05:

(15) $${\mathrm{mAP}}_{0.5:0.95}=\frac{1}{10}\sum_{k=0}^{9}{\mathrm{mAP}}_{0.50+0.05k}$$

Model efficiency is evaluated using the number of parameters, floating-point operations (FLOPs), inference latency (ms/image), and frames per second (FPS). When latency is measured in milliseconds per image, FPS is computed as 1000 divided by the mean inference latency. The parameter count reflects model size, whereas FLOPs approximate the theoretical computational cost of a forward pass. Actual deployment speed can also depend on batch size, warm-up, preprocessing, postprocessing, memory access, framework implementation, and hardware-level operator optimization; therefore, the timing protocol is stated together with each reported result.

## 5. Results and Discussion

### 5.1. Comparison of Convolution Operators

Different convolutional structures were compared to improve feature extraction. The original convolution, diverse branch block (DBB) [22], FDConvC3 [23], and DualConv were incorporated separately into the RT-DETR baseline and evaluated under the same experimental conditions. Detection results are reported in Table 3, and model complexity is compared in Figure 7. The class abbreviations used in Table 3, Table 4 and Table 5 are BS (broken strand), WS (welded strand), BT (bent strand), LS (long scratch), CR (crushed), SS (spaced strand), and DP (deposit).

DualConv provides a favorable balance between detection accuracy and model complexity. Its overall mAP@0.5 is 90.7%, an increase of 1.0 percentage point over the original convolution (89.7%). At the same time, the parameter count decreases from 20.1 M to 16.1 M, and the computational cost decreases from 58.3 to 48.6 GFLOPs.

DBB achieves the highest mAP@0.5 among the convolution variants, at 90.9%, but its parameter count and computational cost increase to 34.7 M and 93.3 GFLOPs, respectively. This gain is therefore accompanied by a substantial increase in model complexity. FDConvC3 reduces the computational cost to 50.9 GFLOPs, but its overall mAP@0.5 decreases to 89.1%, indicating a loss of feature representation in this task.

At the class level, DualConv improves several difficult categories. Its AP@0.5 for bent strand reaches 84.2%, 7.7 percentage points higher than the original convolution, and its AP@0.5 for deposit reaches 98.4%, the highest among the compared methods. DualConv also achieves an mAP@0.5:0.95 of 56.6%, higher than the original convolution, DBB, and FDConvC3, indicating more stable localization at stricter IoU thresholds.

DualConv combines a 3 × 3 grouped convolution with a 1 × 1 pointwise convolution in parallel. This design strengthens local spatial feature extraction while preserving cross-channel interaction and avoids the severe representational loss that can occur in some lightweight convolutional designs.

Considering class-wise accuracy, overall mAP@0.5 and mAP@0.5:0.95, and model complexity, DualConv provides the most suitable trade-off for the present cable defect detection task and is therefore selected as the backbone convolution module.

### 5.2. Comparison of Downsampling Modules

The ability to preserve fine-grained information during repeated downsampling was evaluated by comparing the original downsampling operation, SRFD [24], HWD [25], and the incorporated ContextGuidedDown module in the RT-DETR baseline. All variants were evaluated under the same conditions. Accuracy results are presented in Table 4, and model complexity is shown in Figure 8.

ContextGuidedDown achieves the highest overall mAP@0.5, reaching 90.8%, which is 1.1 percentage points higher than the original downsampling operation (89.7%). Its mAP@0.5:0.95 is 56.3%, slightly above the baseline value of 56.2%. The module has 22.5 M parameters and requires 63.1 GFLOPs, which is higher than some lightweight alternatives but provides a larger accuracy gain.

SRFD reduces information loss through feature rearrangement and requires 19.8 M parameters and 56.2 GFLOPs, but its overall mAP@0.5 is 89.2%. Its performance on fine-grained categories such as bent strand and long scratch is lower than that of the baseline, suggesting limited contextual modeling against complex backgrounds. HWD achieves an overall mAP@0.5 of 90.1% with 19.4 M parameters and 57.0 GFLOPs, showing a reasonable efficiency–accuracy balance; however, its accuracy for bent strand remains below the baseline.

ContextGuidedDown yields particularly clear gains for fine-grained defects. Its AP@0.5 for long scratch is 87.3%, 6.9 percentage points higher than the original downsampling operation, and its result for spaced strand is 91.1%, the highest among the compared methods. The context-guided design therefore helps preserve critical local details and contextual information while reducing feature resolution.

Overall, ContextGuidedDown improves overall accuracy, fine-grained defect detection, and localization stability. Although it increases computational complexity, the accuracy gain is valuable for a defect detection task in which missed small defects can be consequential. It is therefore selected as the downsampling module.

### 5.3. Comparison of High-Level Feature Regulation Strategies

To study the influence of high-level feature processing, Attention [26], AggregatedAtt [27], and DyT were separately introduced into the high-level stage of the RT-DETR baseline and evaluated using the same data split, training settings, and metrics. Attention and AggregatedAtt enhance high-level representations through attention computation or feature aggregation, whereas DyT replaces LayerNorm in the AIFI Transformer encoder and changes the nonlinear feature-regulation mechanism. The results are presented in Table 5, and model complexity is compared in Figure 9.

DyT achieves the best results among these three high-level strategies. Its overall mAP@0.5 reaches 90.6%, 0.9 percentage points above the baseline (89.7%), and its mAP@0.5:0.95 increases from 56.2% to 56.5%. The model contains 20.0 M parameters and requires 58.3 GFLOPs, which is essentially unchanged from the baseline.

Adding Attention increases the overall mAP@0.5 from 89.7% to 89.8% but decreases mAP@0.5:0.95 from 56.2% to 55.7%. The parameter count and computational cost increase to 20.8 M and 58.6 GFLOPs. Although the deposit category reaches 99.1%, accuracy decreases for welded strand, bent strand, and crushed, indicating that the overall gain is limited and inconsistent across categories.

AggregatedAtt uses a more complex attention aggregation structure and increases the parameter count and computational cost to 23.5 M and 64.0 GFLOPs. Its overall mAP@0.5 and mAP@0.5:0.95 decrease to 88.3% and 53.2%, respectively. Although it achieves 91.4% and 99.4% for broken strand and welded strand, its results for bent strand and long scratch fall to 67.5% and 77.0%. Thus, the additional high-level attention complexity does not provide a stable benefit in this task.

DyT improves several weaker categories of the baseline. Its AP@0.5 for bent strand rises from 76.5% to 79.7%, and that for long scratch rises from 80.4% to 83.0%. Results for spaced strand and deposit increase from 89.1% and 95.4% to 90.0% and 98.1%, respectively, while welded strand remains unchanged at 98.4%. Accuracy for broken strand and crushed decreases by 1.7 percentage points in each case, showing that the gain is not uniform across all categories; nevertheless, both overall mAP metrics improve.

At the category level, replacing LayerNorm with DyT increases AP@0.5 for bent strand by 3.2 percentage points and for long scratch by 2.6 percentage points, whereas the effects on the other categories are mixed. This indicates that the benefit of DyT is category-dependent rather than uniform. One possible explanation is that DyT changes the numerical regulation of high-level AIFI features through a learnable input scale, element-wise tanh compression, and channel-wise affine transformation, while leaving the original self-attention mechanism unchanged. Such learnable feature regulation may provide a more suitable response range for difficult defect representations that are relatively weak in the baseline. However, this interpretation should be regarded as a plausible mechanism rather than a causal conclusion, because no feature-attribution or class-specific representation analysis was conducted in this study. Moreover, bent strand is the least frequent category in CableInspect-AD, so class imbalance may also contribute to the observed performance variation. The larger gains for bent strand and long scratch in the complete DCGD-RTDETR model (7.0 and 5.8 percentage points, respectively) should therefore be interpreted as the combined effect of all three modifications rather than as evidence of DyT alone.

Attention provides only a marginal overall gain, and AggregatedAtt decreases accuracy while increasing model complexity. DyT, by contrast, raises mAP@0.5 and mAP@0.5:0.95 to 90.6% and 56.5% with essentially unchanged complexity. It is therefore selected as the high-level feature-regulation strategy in AIFI.

### 5.4. Ablation Study

Ablation experiments were conducted using RT-DETR as the baseline. DualConv, ContextGuidedDown (CGDown), and DyT were introduced individually and in combination. All experiments used the same dataset split, training settings, and evaluation metrics. The results are reported in Table 6.

Each individual module improves mAP@0.5 to some extent. DualConv raises mAP@0.5 from 89.7% to 90.7% while reducing the number of parameters from 20.1 M to 16.1 M and the computational cost from 58.3 to 48.6 GFLOPs. CGDown increases mAP@0.5 to 90.8%, although the parameter count and computational cost rise to 22.5 M and 63.1 GFLOPs, respectively. Replacing LayerNorm in AIFI with DyT increases mAP@0.5 to 90.6% while leaving model complexity nearly unchanged.

For the two-module combinations, DualConv + CGDown achieves an mAP@0.5 of 91.7% with 18.9 M parameters and 53.9 GFLOPs. DualConv + DyT reaches 91.1% mAP@0.5 while requiring only 15.9 M parameters and 47.3 GFLOPs, giving the lowest complexity among the evaluated ablation variants. CGDown + DyT obtains 91.6% mAP@0.5 with 22.3 M parameters and 61.7 GFLOPs. The retained inference-only timing records for DualConv + DyT and CGDown + DyT are 8.740 and 9.681 ms/image, corresponding to 114.42 and 103.30 FPS, respectively. Their AP@0.75 values are 68.79% and 63.73%. These results show that the effects of the modules are complementary but not simply additive, because different combinations produce different accuracy–complexity trade-offs.

When all three modules are combined, DCGD-RTDETR achieves the highest mAP@0.5 of 92.4%, which is 2.7 percentage points above the RT-DETR baseline. The complete model contains 18.6 M parameters and requires 53.4 GFLOPs, corresponding to reductions of approximately 7.5% and 8.4%, respectively, relative to the baseline. Although DualConv + DyT is more compact, the full combination provides the best detection accuracy while remaining less complex than the baseline.

The three modules act at different stages of the network. DualConv improves local feature extraction and cross-channel interaction in the backbone while reducing complexity. CGDown preserves local details and models contextual information during downsampling, but its individual use increases computational cost. DyT replaces LayerNorm in AIFI and provides learnable nonlinear regulation of high-level features. The complete ablation over all eight possible combinations therefore confirms that the final configuration provides the most favorable balance between detection accuracy and model complexity for this task.

Accordingly, DualConv, ContextGuidedDown, and DyT are combined to form the complete DCGD-RTDETR model.

### 5.5. Comparison with Representative Object Detectors

DCGD-RTDETR was compared with representative object detectors, including YOLOv11n, YOLOv8n, Faster R-CNN, and the RT-DETR baseline. All compared models used the same CableInspect-AD training, validation, and test partitions, the same input size, and the same held-out test-set evaluation protocol. Architecture-specific optimization settings were retained and are documented in Table 2 rather than being artificially forced to be identical across detector families. The RT-DETR baseline used the R18 configuration, while Faster R-CNN used a ResNet-50-FPN backbone with COCO-pretrained weights. The detection results are summarized in Table 7, and the corresponding model-complexity comparison is presented in Figure 10.

DCGD-RTDETR achieves the highest overall mAP@0.5 among the compared models, reaching 92.4%, compared with 84.1% for YOLOv11n, 79.4% for YOLOv8n, 79.8% for Faster R-CNN, and 89.7% for the RT-DETR baseline. At the more stringent mAP@0.5:0.95 metric, DCGD-RTDETR achieves 57.1%, which is slightly higher than the RT-DETR baseline (56.2%) and higher than YOLOv11n (49.2%), YOLOv8n (47.8%), and Faster R-CNN (44.7%). These results indicate that DCGD-RTDETR provides strong defect-detection accuracy while maintaining lower model complexity than the RT-DETR baseline. At AP@0.75, YOLOv11n, YOLOv8n, Faster R-CNN, RT-DETR, and DCGD-RTDETR achieve 50.7%, 47.9%, 47.6%, 61.7%, and 63.7%, respectively.

YOLOv11n and YOLOv8n have clear advantages in model compactness, requiring only 2.6 M/6.3 GFLOPs and 3.0 M/8.1 GFLOPs, respectively. However, their overall mAP@0.5 values are lower than those of RT-DETR and DCGD-RTDETR. YOLOv11n reaches 65.5% and 64.7% AP@0.5 on bent strand and long scratch, respectively, while YOLOv8n reaches 65.1% and 64.2%. These results suggest that the two lightweight YOLO variants remain less effective than the RT-DETR-based models for these subtle defect categories under the present experimental setting.

Faster R-CNN, implemented with a ResNet-50-FPN backbone and COCO-pretrained weights, achieves an overall mAP@0.5 of 79.8%, an AP@0.75 of 47.6%, and an mAP@0.5:0.95 of 44.7% on the test set. Its per-class AP@0.5 values are 74.4%, 96.0%, 69.1%, 76.8%, 83.0%, 70.0%, and 89.6% for broken strand, welded strand, bent strand, long scratch, crushed, spaced strand, and deposit, respectively. The model contains 41.4 M parameters and requires approximately 182.2 GFLOPs under the adopted complexity-estimation protocol. Using batch size 1 on the RTX 3090 with 50 warm-up images, the mean inference latency is 14.819 ms/image, corresponding to 67.48 FPS. These results show that Faster R-CNN has substantially greater computational complexity than the lightweight one-stage detectors and the RT-DETR-based models evaluated in this study, while achieving lower overall detection accuracy on this dataset.

The RT-DETR baseline already performs strongly on this task, with an overall mAP@0.5 of 89.7%, 20.1 M parameters, and 58.3 GFLOPs. DCGD-RTDETR incorporates DualConv, ContextGuidedDown, and DyT to improve detection performance while reducing model complexity.

Compared with RT-DETR, DCGD-RTDETR increases the overall mAP@0.5 by 2.7 percentage points. The largest improvements occur for bent strand and long scratch, which increase by 7.0 and 5.8 percentage points, respectively. Broken strand, spaced strand, and deposit also improve; welded strand remains unchanged, whereas crushed shows a slight decrease. Meanwhile, the parameter count decreases from 20.1 M to 18.6 M, and the computational cost decreases from 58.3 GFLOPs to 53.4 GFLOPs.

A qualitative comparison between RT-DETR and DCGD-RTDETR is shown in Figure 11.

Overall, DCGD-RTDETR provides a favorable accuracy–complexity trade-off for cable defect detection. It achieves the highest mAP@0.5 among the compared detectors and slightly improves mAP@0.5:0.95 over the RT-DETR baseline while reducing both parameter count and computational cost. Compared with Faster R-CNN, DCGD-RTDETR also achieves substantially higher detection accuracy with a considerably smaller model. Its reported mAP@0.5:0.95 of 57.1% is also higher than those of YOLOv11n (49.2%) and YOLOv8n (47.8%). Nevertheless, the repeated-run AP@0.75 analysis indicates that localization accuracy under stricter overlap criteria remains an area for further improvement. Because the available timing records were obtained through different evaluation pipelines, they are interpreted as practical reference values rather than as a strictly controlled cross-framework speed ranking.

### 5.6. External Validation on RF100 Cable Damage

Additional experiments were performed on the RF100 Cable Damage dataset, whose category definitions, sample distribution, and target appearance differ from those of CableInspect-AD. YOLOv11n, RT-DETR, and DCGD-RTDETR were trained and tested using the same data split, training settings, and evaluation metrics within this dataset. The results are reported in Table 8.

DCGD-RTDETR achieves the highest mAP@0.5 on RF100 Cable Damage, reaching 91.9%. This is 1.3 percentage points higher than RT-DETR and 3.6 percentage points higher than YOLOv11n. Because all models were trained and evaluated on RF100, this experiment should be interpreted as external validation under a different dataset distribution rather than zero-shot cross-dataset generalization. The result shows that, within this two-category external dataset, the proposed architecture retains an advantage over the RT-DETR baseline in mAP@0.5 while using fewer parameters and FLOPs. A qualitative comparison is shown in Figure 12.

YOLOv11n has the lowest complexity, with 2.6 M parameters and 6.3 GFLOPs, but its mAP@0.5 is lower than those of RT-DETR and DCGD-RTDETR. RT-DETR requires 19.9 M parameters and 56.9 GFLOPs, whereas DCGD-RTDETR reduces these values to 18.5 M and 52.1 GFLOPs while improving mAP@0.5.

For mAP@0.5:0.95, DCGD-RTDETR reaches 46.2%, 0.8 percentage points above RT-DETR (45.4%). YOLOv11n obtains a slightly higher value of 46.6%, although its mAP@0.5 is lower. Thus, YOLOv11n shows competitive localization quality across the averaged IoU thresholds, whereas DCGD-RTDETR provides the highest detection result at IoU = 0.5.

In summary, DCGD-RTDETR achieves the highest mAP@0.5 on RF100 Cable Damage while using fewer parameters and FLOPs than RT-DETR. This result provides additional evidence that the proposed modifications can remain effective under a different cable-damage data distribution. However, RF100 Cable Damage contains only two annotated categories, so these results should not be interpreted as evidence of broad cross-dataset or multi-category generalization.

### 5.7. Failure-Case and Error Analysis

To further investigate the limitations of DCGD-RTDETR, a diagnostic error analysis was conducted on the CableInspect-AD test set using a confidence threshold of 0.25 and an IoU matching threshold of 0.5. Under this setting, among the 393 ground-truth defect instances, 358 were correctly matched, while 35 false negatives and 65 false positives were observed, corresponding to an overall recall of 91.1%. The per-class error statistics are summarized in Table 9. This diagnostic analysis is intended to characterize representative detection failures and is complementary to the standard AP-based evaluation reported in the preceding sections.

The error distribution is clearly non-uniform across categories. Bent strand exhibits the lowest recall, at 77.3%, with five missed detections among 22 ground-truth instances. Spaced strand contributes the largest absolute number of errors, with 20 false negatives and 31 false positives. In contrast, all 61 deposit instances are detected under the adopted matching criterion, with only one additional false-positive prediction. These results indicate that the remaining detection errors are concentrated in several challenging categories rather than being evenly distributed over the dataset.

The error pattern should also be interpreted together with the class distribution. Bent strand is the least frequent category in the dataset, with 163 instances, and it also shows the lowest recall in the diagnostic analysis. In contrast, spaced strand is the most frequent category, with 1309 instances, and therefore contributes the largest absolute number of false positives and false negatives. These observations do not establish that class frequency alone causes the errors, because defect morphology and background ambiguity also affect detection difficulty; however, they indicate that class imbalance is relevant when interpreting category-level stability and remains an important limitation of the current dataset.

Representative failure cases are shown in Figure 13. In Figure 13a, the bent-strand defect is identified, but the predicted bounding box is only partially aligned with the ground-truth region and the confidence is relatively low, indicating localization uncertainty for this subtle defect pattern. Figure 13b illustrates a multi-defect scene in which several defects are correctly detected, while additional false-positive predictions and imperfect localization are also present. In Figure 13c, the spaced-strand defect is detected but the predicted box deviates noticeably from the ground-truth annotation, and an additional false-positive broken-strand prediction is produced. Figure 13d shows false-positive spaced-strand detections in a region without a corresponding ground-truth defect, suggesting that regular cable-strand texture can occasionally resemble local spacing anomalies.

### 5.8. Statistical Robustness Across Random Seeds

To assess the robustness of the reported results to training stochasticity, the RT-DETR baseline and DCGD-RTDETR were each trained four times using the same random seeds (1, 2, 3, and 4), while all other training settings and the train/validation/test split were kept unchanged. Performance was evaluated on the same test set. Table 10 reports the result of every individual run together with the mean ± sample standard deviation (SD) and the 95% confidence interval (CI). Because the same seed values were used for the two models, a two-sided paired *t*-test was used for paired comparison. Given the limited number of repeated runs (n = 4), the inferential statistics are interpreted cautiously. These repeated-run results are reported separately from the single-run results in the main comparison tables and are intended specifically to assess robustness to random variation.

Across the four paired runs, DCGD-RTDETR achieved an mAP@0.5 of 92.05 ± 0.34%, compared with 90.98 ± 1.10% for the RT-DETR baseline. The paired mean difference was +1.07 percentage points (95% CI: −0.28 to 2.42 percentage points), and the paired *t*-test yielded *p* = 0.086. Thus, the repeated-run results show a higher mean mAP@0.5 and substantially lower run-to-run variability for DCGD-RTDETR, while the paired difference does not reach statistical significance at the 0.05 level. DCGD-RTDETR achieved a higher mAP@0.5 than the corresponding baseline run under all four paired seeds.

For mAP@0.5:0.95, the baseline and DCGD-RTDETR achieved 57.99 ± 1.37% and 58.19 ± 0.73%, respectively, corresponding to a paired mean difference of +0.20 percentage points (95% CI: −2.00 to 2.40; *p* = 0.791). In contrast, AP@0.75 decreased from 65.01 ± 1.43% for the baseline to 63.26 ± 1.11% for DCGD-RTDETR, with a paired mean difference of −1.75 percentage points (95% CI: −2.51 to −0.99; *p* = 0.005). These results indicate that the main repeated-run advantage of DCGD-RTDETR lies in its higher and more stable mAP@0.5, whereas localization performance at a stricter IoU threshold remains a limitation and should be improved in future work. The small number of repeated runs should also be considered when interpreting the statistical tests.

### 5.9. Supplementary Inference-Efficiency Measurements

To complement the accuracy and robustness analyses, inference-efficiency measurements are reported in Table 11. The RT-DETR baseline and DCGD-RTDETR were re-evaluated using the same RT-DETR evaluation pipeline on the NVIDIA GeForce RTX 3090, allowing a direct within-framework comparison of inference-only latency and throughput. The retained DualConv + DyT and CGDown + DyT records use the same inference-only timing definition. YOLOv11n was measured separately using the Ultralytics 8.3.9 evaluation pipeline, which reports an inference-only latency of 0.967 ms/image (1034.33 FPS). Faster R-CNN was timed separately with batch size 1 after 50 warm-up images on the same GPU. Because the YOLOv11n and Faster R-CNN timing pipelines are not identical to the RT-DETR-based pipeline, their latency values are interpreted as practical references rather than as strictly controlled cross-framework benchmarks.

Under the same RT-DETR timing pipeline, the baseline requires 6.847 ms/image for inference (146.05 FPS), whereas DCGD-RTDETR requires 8.930 ms/image (111.99 FPS). Thus, although the proposed model reduces the parameter count and theoretical FLOPs in the main complexity analysis, this reduction does not translate into lower wall-clock inference latency in the present implementation. Practical latency depends not only on FLOPs but also on operator implementation, memory access, kernel scheduling, and hardware utilization; no kernel-level profiling was performed here, so the source of the additional latency is not attributed to any individual module. Nevertheless, DCGD-RTDETR maintains an inference throughput above 100 FPS while improving the main single-run mAP@0.5 from 89.7% to 92.4%. In the separate Ultralytics pipeline, YOLOv11n requires 0.967 ms/image for inference, corresponding to 1034.33 FPS; because its framework and timing pipeline differ, this value is reported only as a practical reference rather than a strict cross-framework speed ranking. The DualConv + DyT and CGDown + DyT configurations also exceed 100 FPS in the retained inference-only records, while Faster R-CNN reaches 67.48 FPS under its separate batch-1 timing protocol.

## 6. Conclusions

This study addresses small target size, subtle morphological differences, complex background interference, and high computational cost in cable defect detection for transmission-line inspection. Using RT-DETR as the baseline, a modified detector named DCGD-RTDETR is developed and evaluated on CableInspect-AD and the RF100 Cable Damage external validation dataset.

The original RT-DETR is modified to address insufficient feature representation, information loss during downsampling, and limited regulation of high-level semantic features. DualConv, ContextGuidedDown, and DyT are incorporated to improve low-level feature extraction, preserve information during downsampling, and regulate high-level AIFI features, respectively. DyT replaces LayerNorm in the Transformer encoder and performs a learnable nonlinear transformation without computing feature means and variances.

On CableInspect-AD, DCGD-RTDETR achieves an mAP@0.5 of 92.4%, 2.7 percentage points higher than the original RT-DETR in the main single-run comparison. The parameter count decreases from 20.1 M to 18.6 M, and the computational cost decreases from 58.3 to 53.4 GFLOPs. Across four paired random-seed runs, DCGD-RTDETR also shows a higher mean mAP@0.5 and lower variability than the baseline (92.05 ± 0.34% vs. 90.98 ± 1.10%), although the paired improvement does not reach the 0.05 significance level. The lower AP@0.75 observed in the repeated runs indicates that stricter-IoU localization accuracy remains an area for further improvement.

The proposed method therefore provides a favorable detection-accuracy and model-complexity trade-off, with its clearest advantage appearing at IoU = 0.5. It improves several difficult categories, particularly bent strand and long scratch, while reducing the parameter count and theoretical computational cost relative to the RT-DETR baseline. However, the same-pipeline RTX 3090 timing experiment shows that inference latency increases from 6.847 to 8.930 ms/image (146.05 to 111.99 FPS), so the complexity reduction should not be interpreted as a wall-clock speed advantage under the current implementation. The strict-IoU results likewise show that the accuracy improvement should not be interpreted as uniform improvement in localization quality at all overlap thresholds.

This study has several limitations. The scale of the datasets, the range of operating conditions, and the diversity of real inspection scenes remain limited, so performance under additional complex conditions requires further validation. Because the datasets used in this study consist primarily of close-range cable images, the present experiments do not directly validate performance in high-altitude UAV inspection scenarios. In addition, the RF100 Cable Damage external dataset contains only two annotated categories; broader multi-category external validation is therefore still required. Some defect categories contain relatively few samples, and class imbalance may affect detection stability. Very small targets, severe occlusion, background textures similar to defects, and blurred images may still lead to false or missed detections.

Future work will extend cable defect data to more scenes and operating conditions and investigate data augmentation and class-balanced learning for underrepresented categories. Model pruning, knowledge distillation, and quantization will also be explored to improve deployment on edge devices such as unmanned aerial vehicles and inspection robots. Integrating multimodal information, temporal image analysis, and defect-severity assessment may further improve the practicality of intelligent power-equipment inspection and condition assessment.

## Figures and Tables

**Figure 1:**
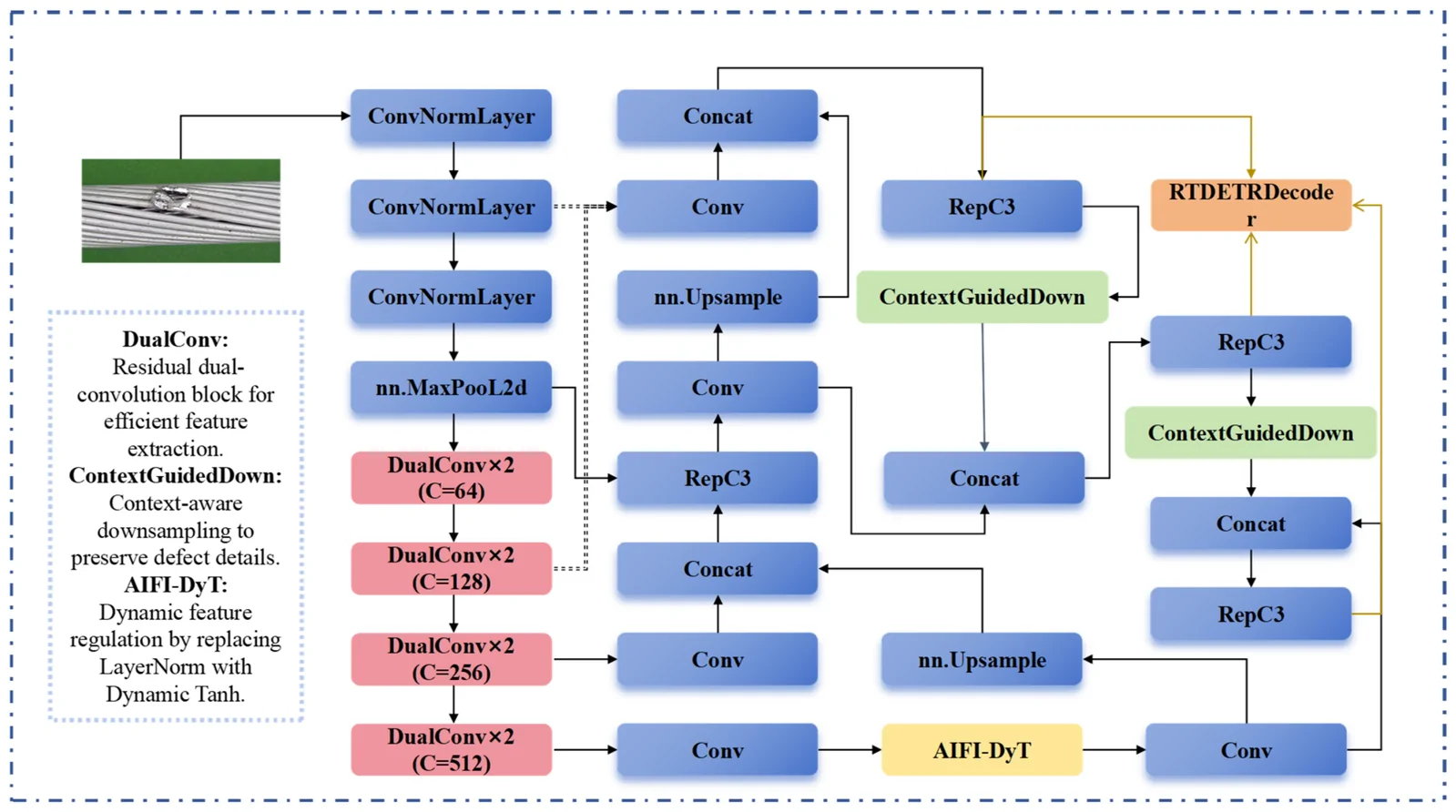
Overall architecture of DCGD-RTDETR.

**Figure 2:**
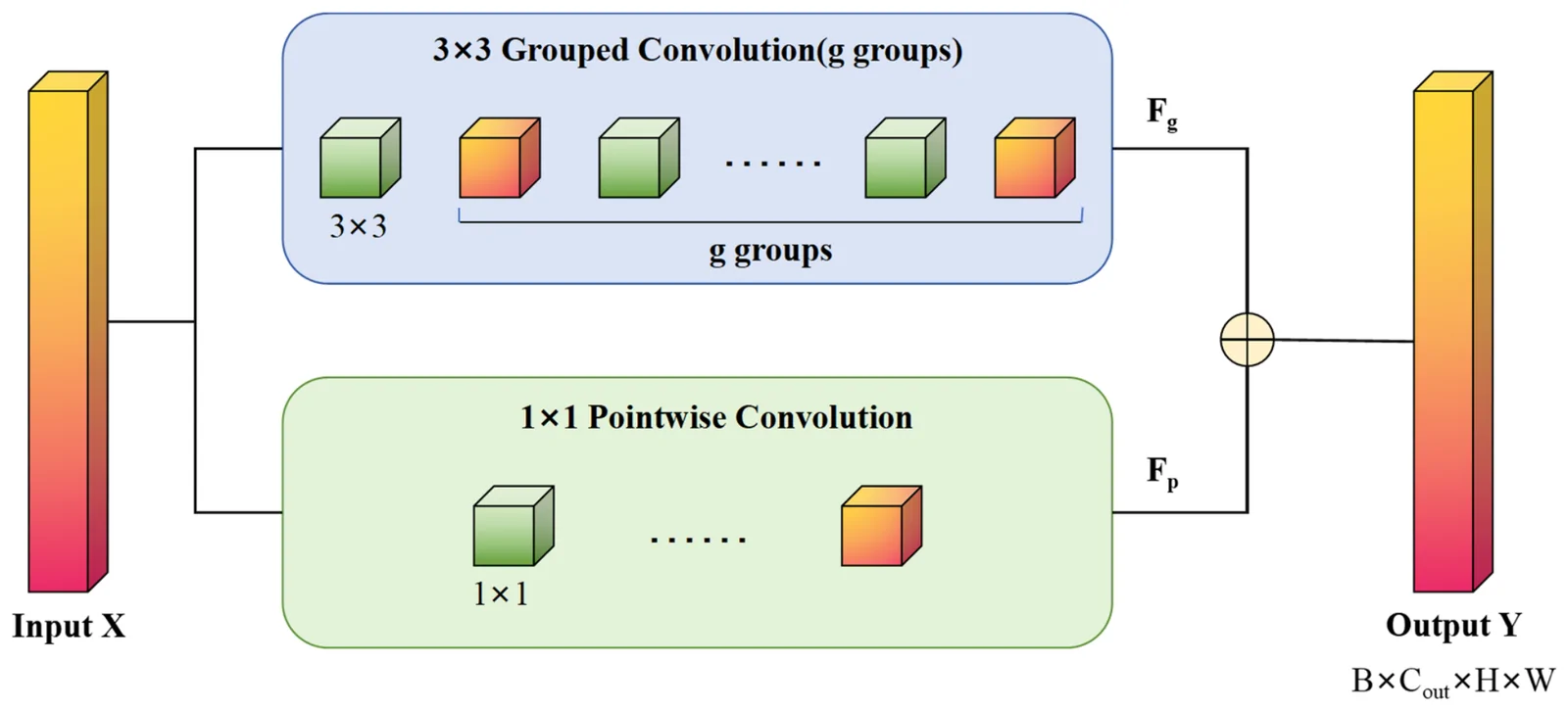
Structure of DualConv.

**Figure 3:**
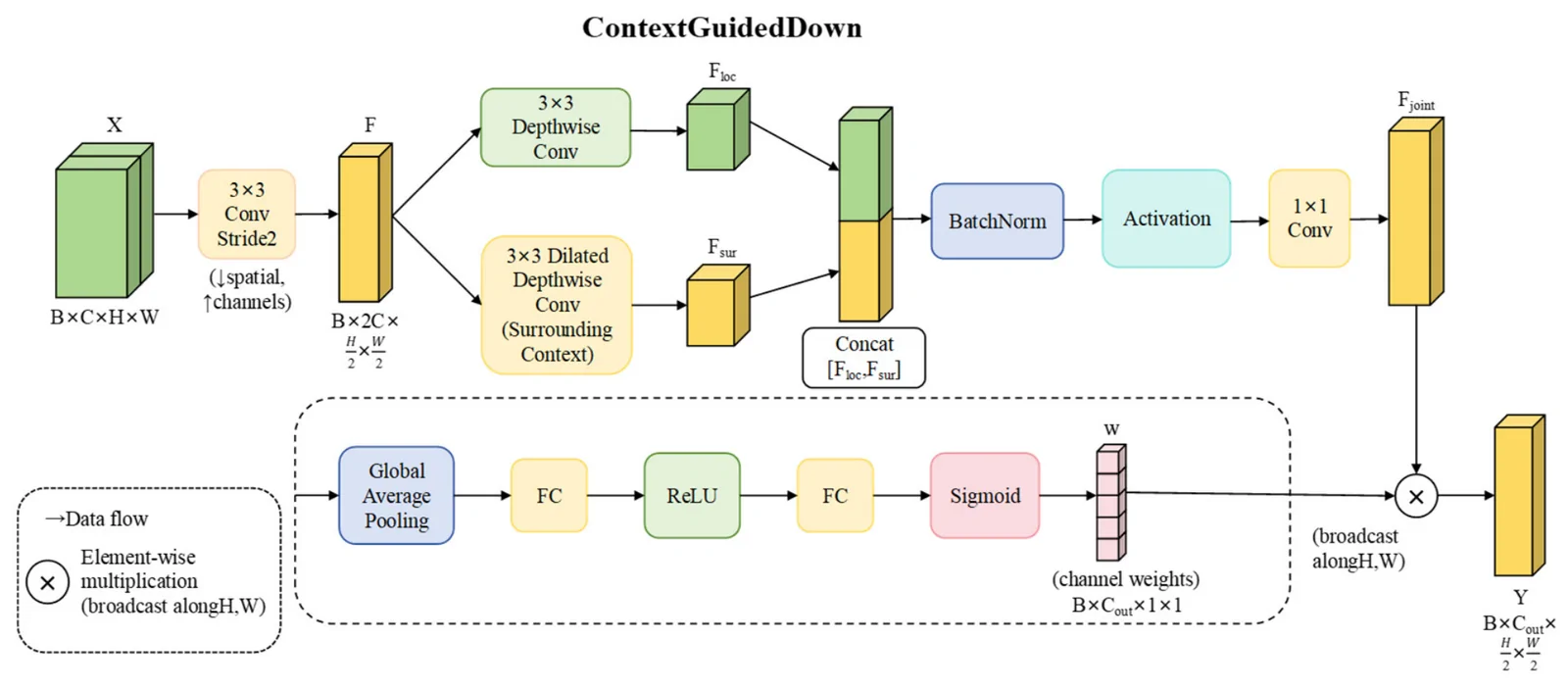
Structure of ContextGuidedDown.

**Figure 4:**
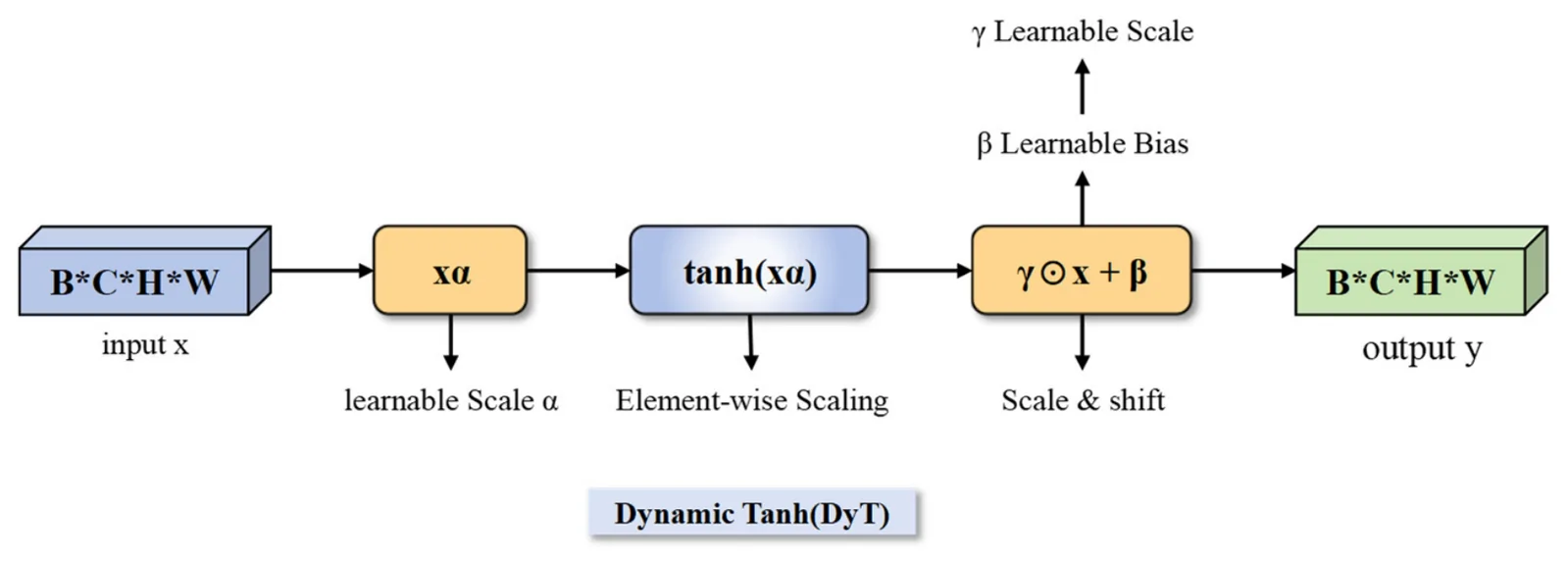
Structure of Dynamic Tanh (DyT).

**Figure 5:**
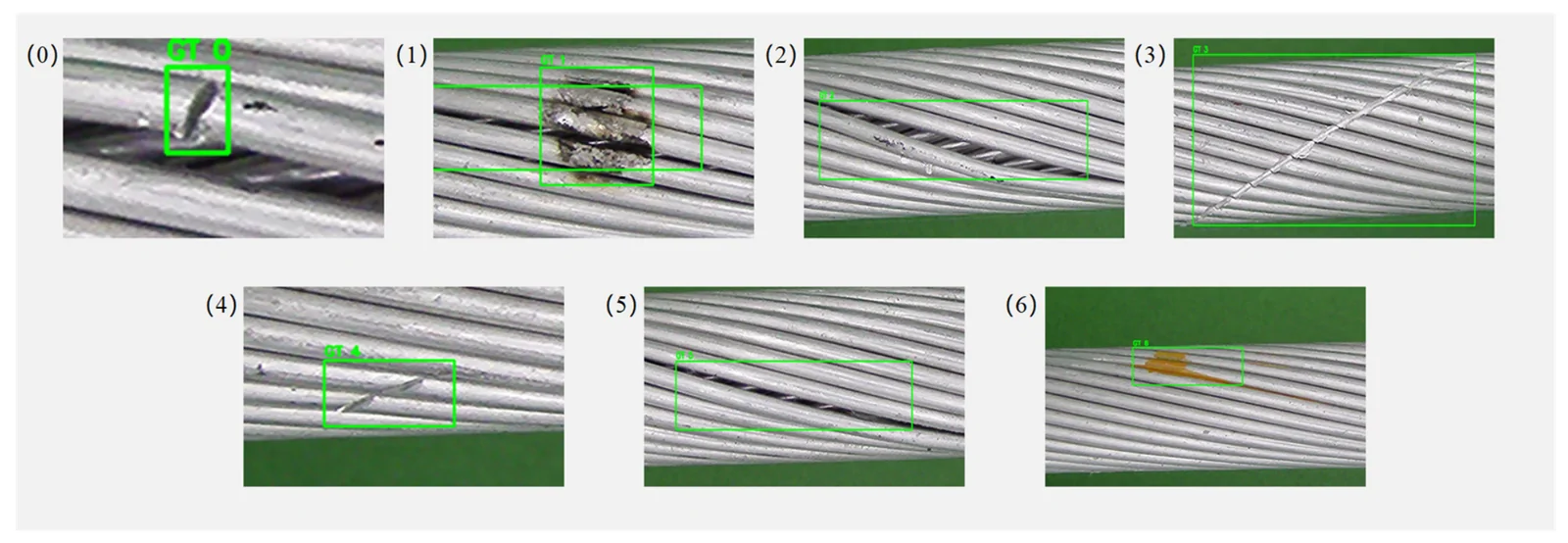
Examples of seven cable defect categories: (**0**) broken strand; (**1**) welded strand; (**2**) bent strand; (**3**) long scratch; (**4**) crushed; (**5**) spaced strand; and (**6**) deposit. Green boxes indicate annotated defect regions.

**Figure 6:**
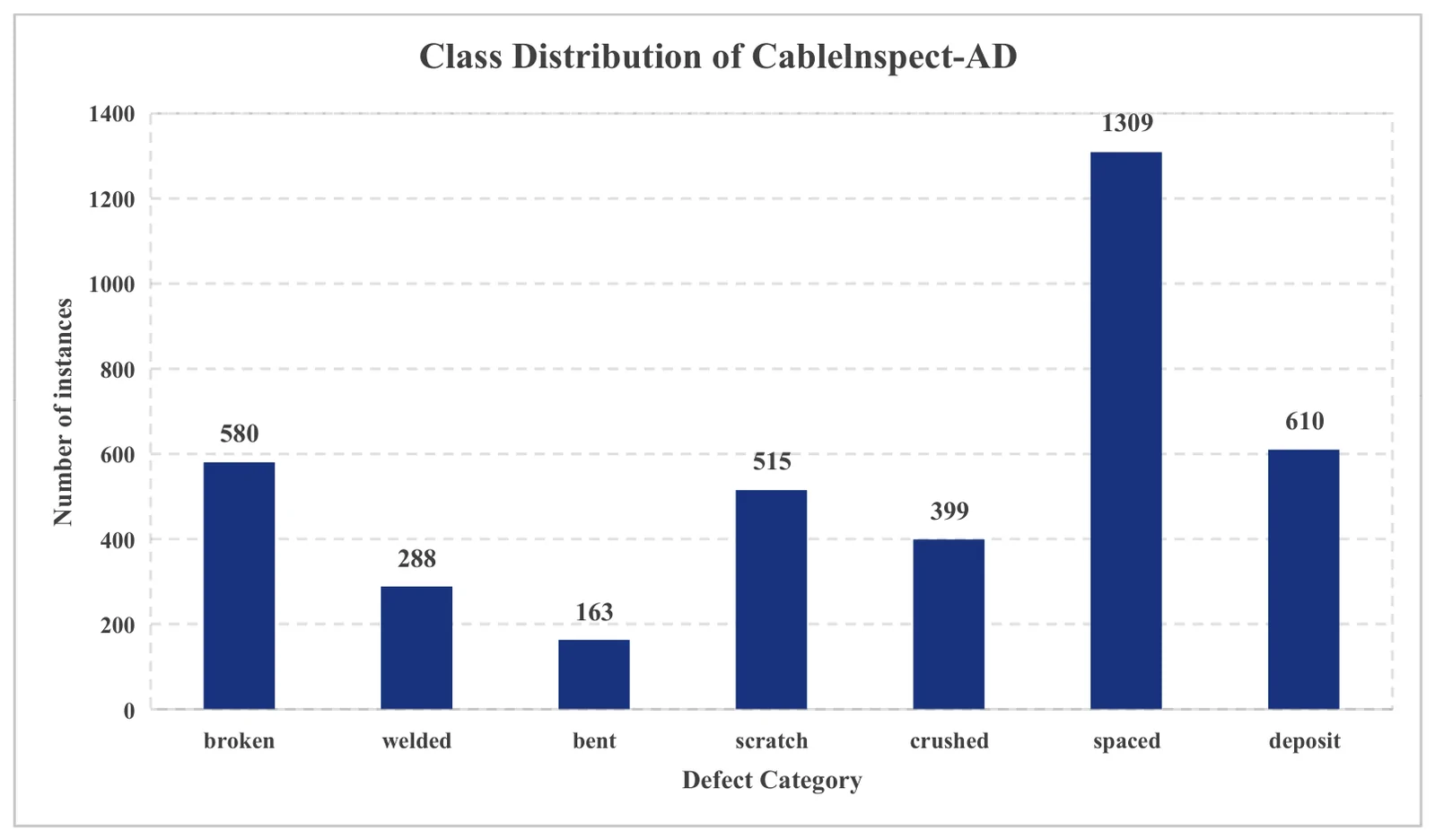
Class distribution of the CableInspect-AD dataset.

**Figure 7:**
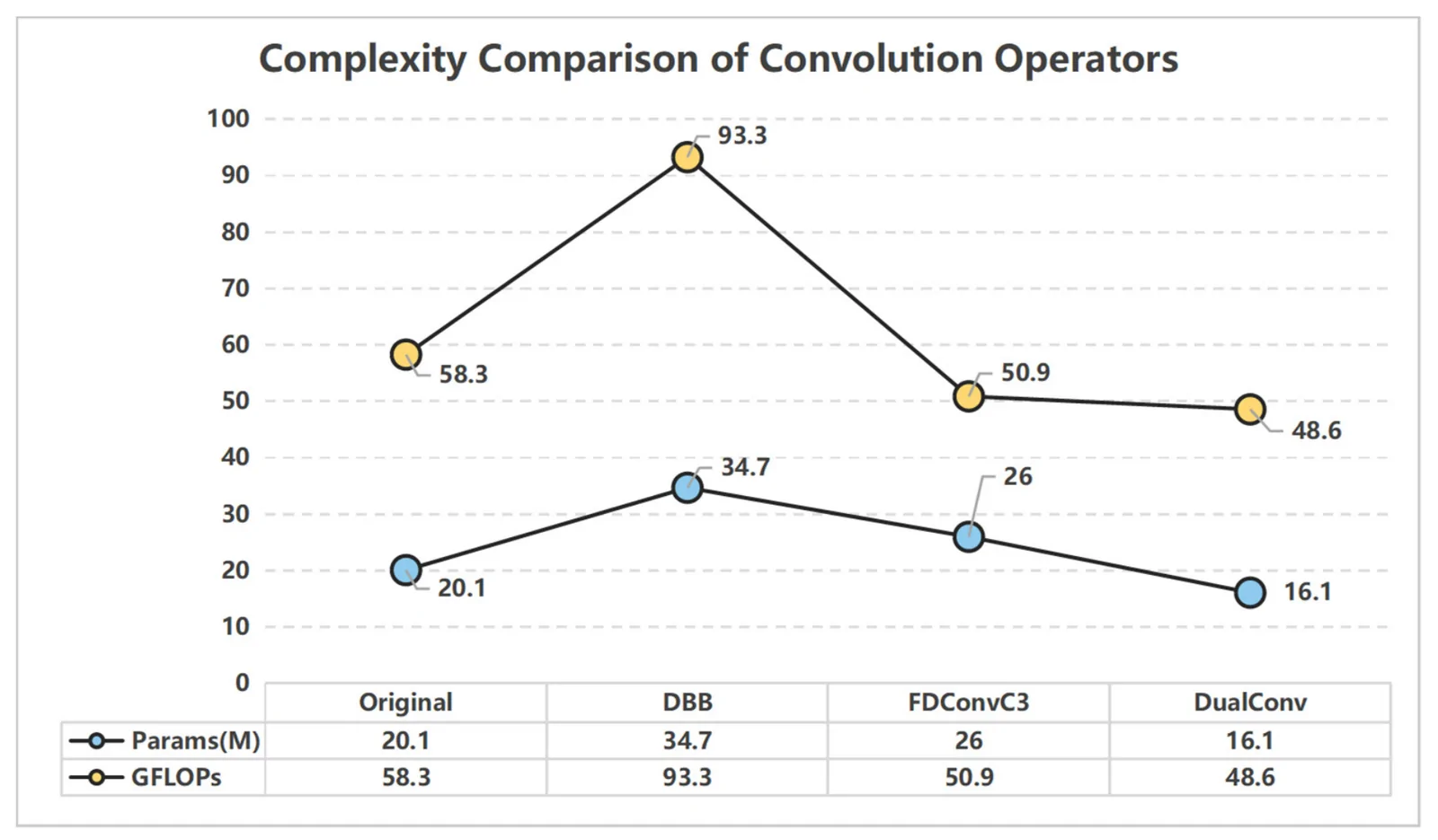
Parameter count and computational cost of different convolution operators.

**Figure 8:**
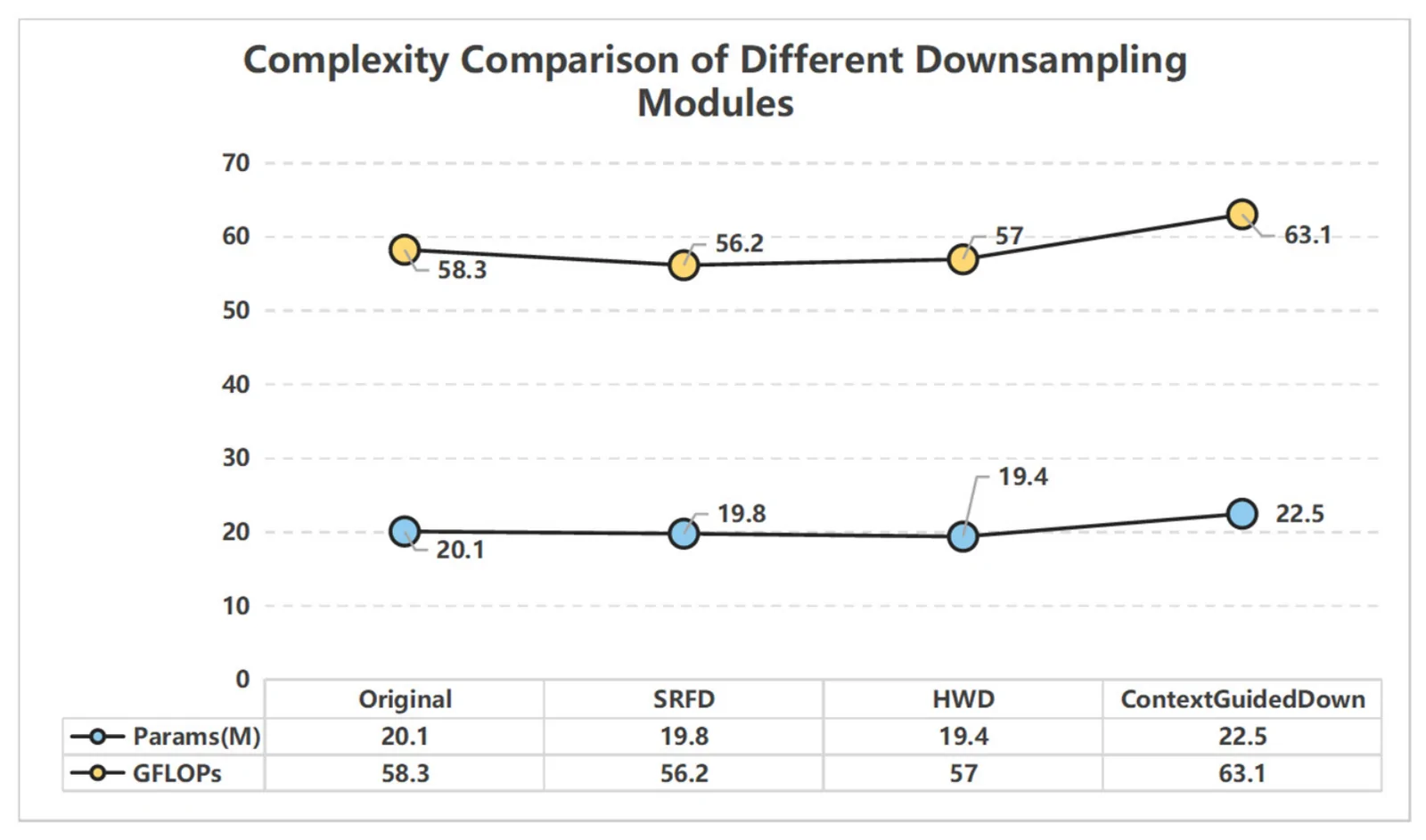
Model complexity of different downsampling modules.

**Figure 9:**
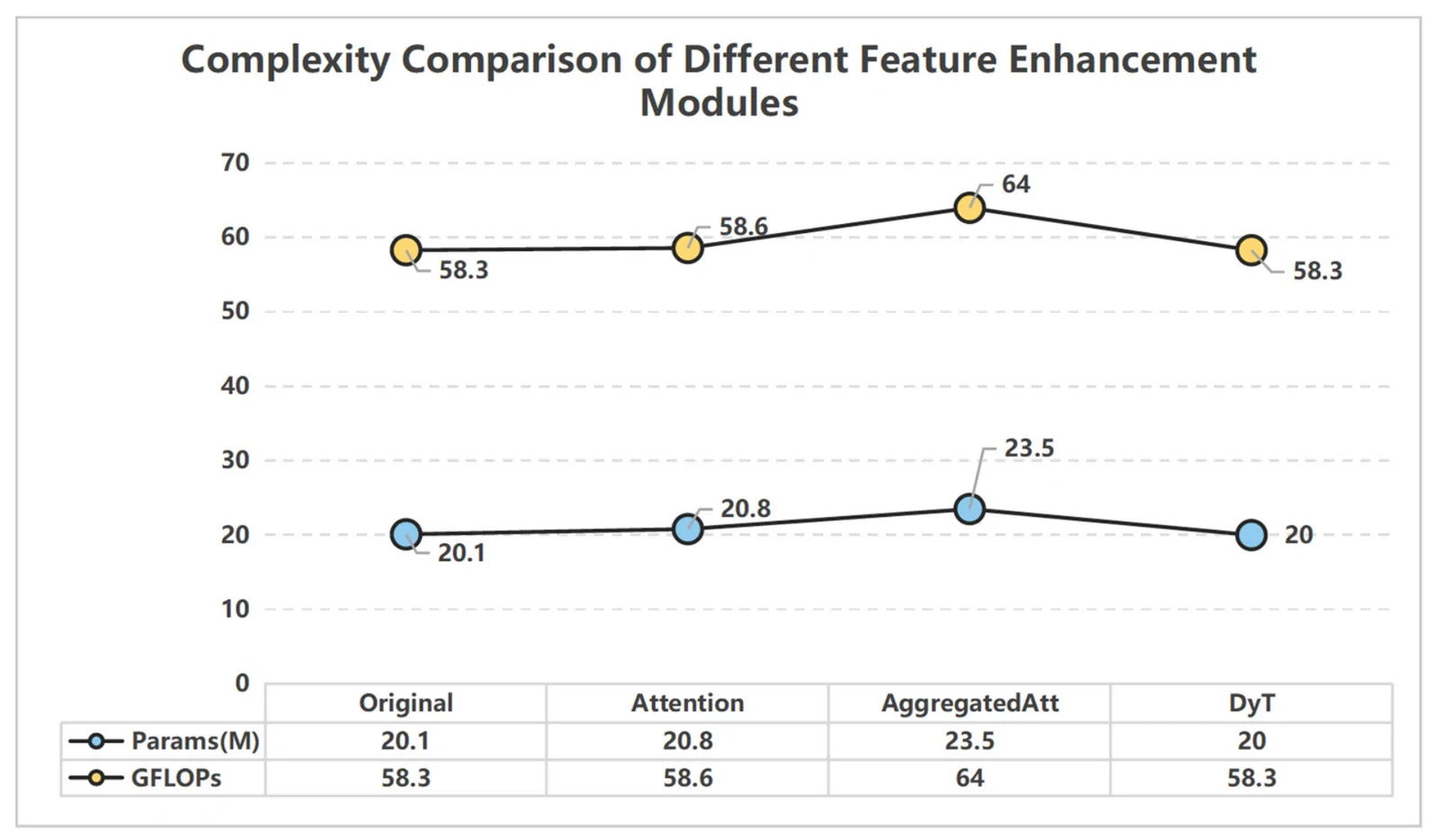
Model complexity of different high-level feature regulation strategies.

**Figure 10:**
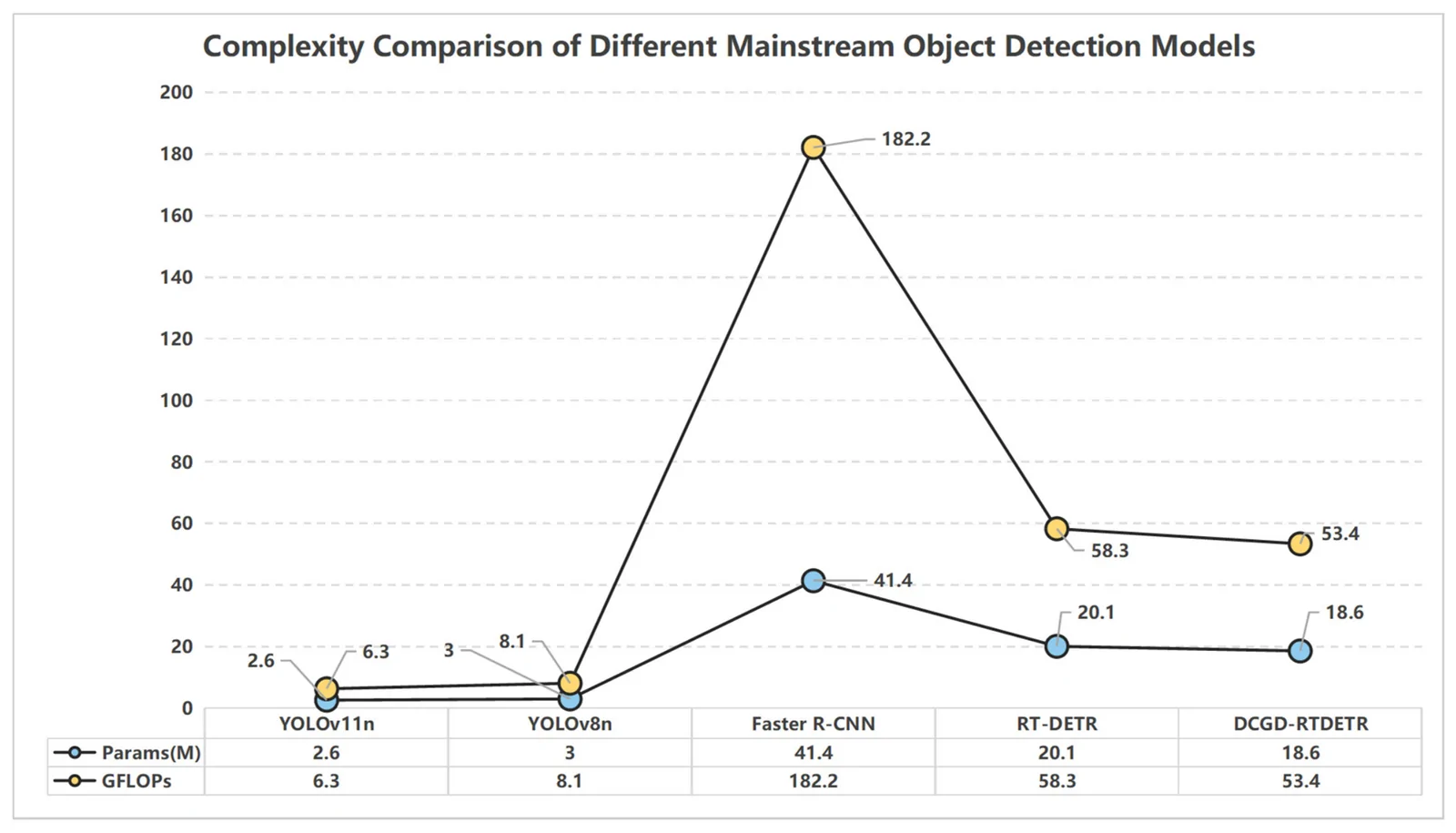
Complexity comparison of representative object detectors.

**Figure 11:**
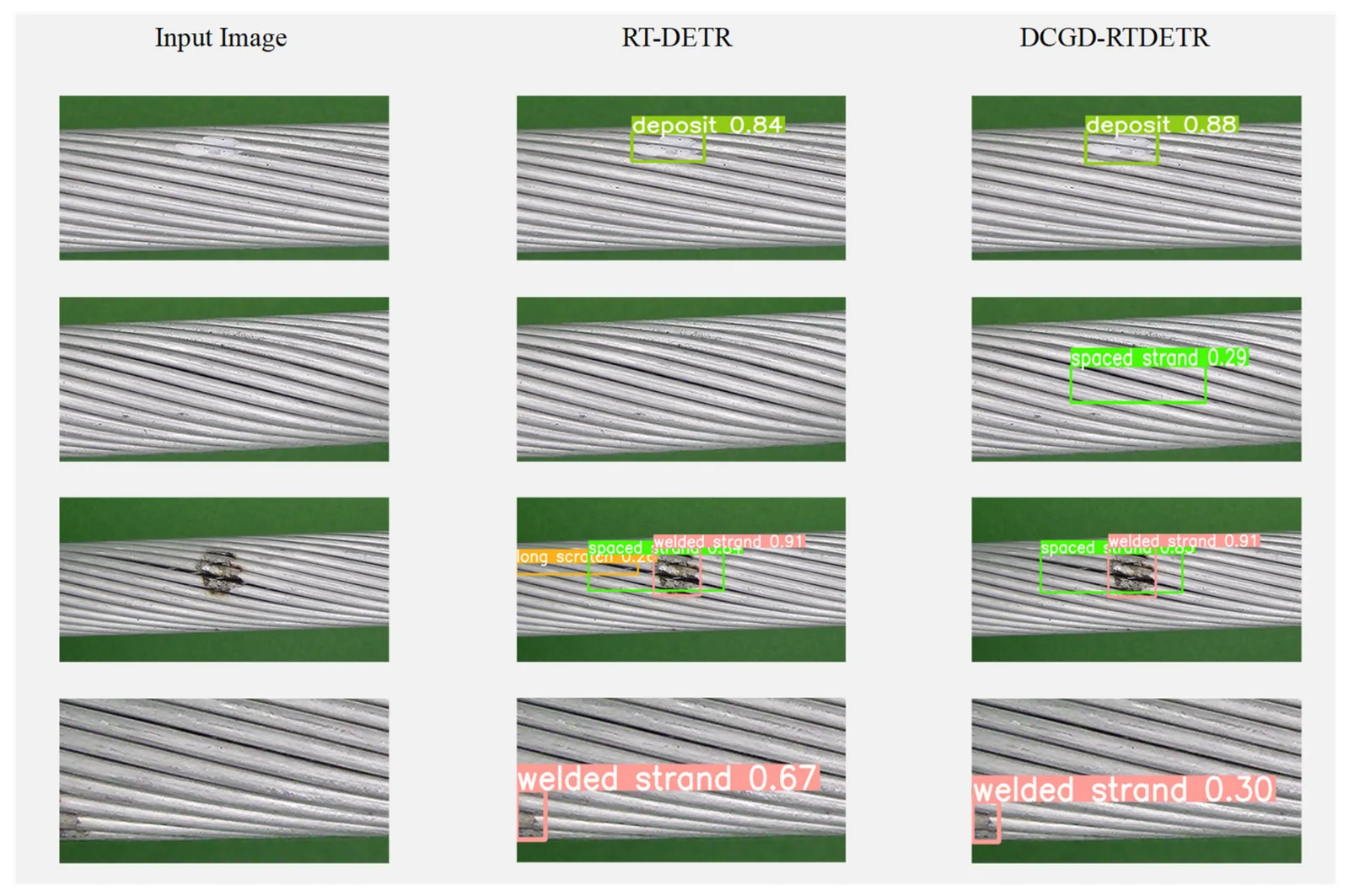
Qualitative comparison between RT-DETR and DCGD-RTDETR.

**Figure 12:**
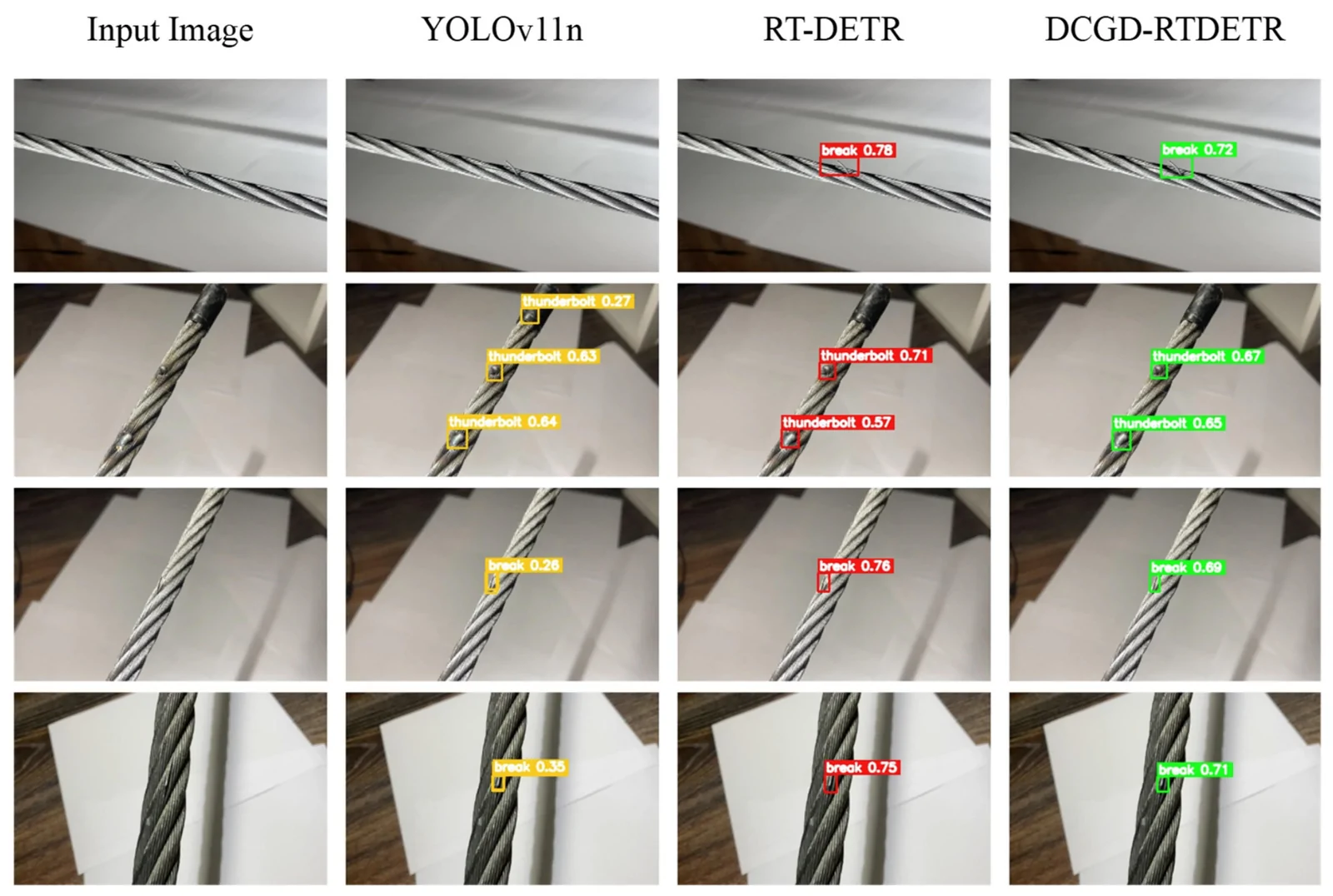
Qualitative comparison on the RF100 Cable Damage dataset.

**Figure 13:**
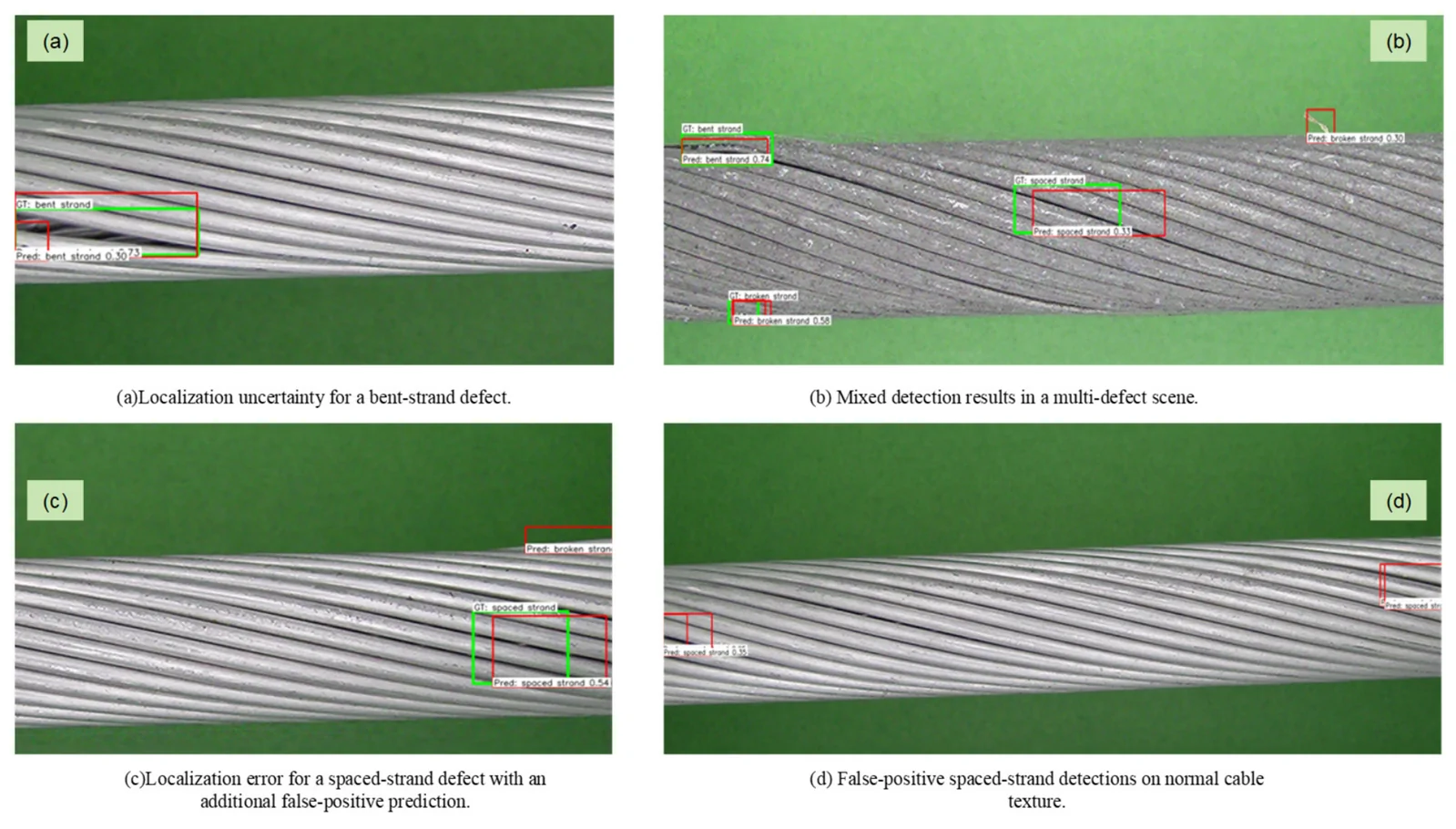
Representative failure cases of DCGD-RTDETR on the CableInspect-AD test set. Green boxes denote ground-truth annotations, while red boxes denote model predictions. (**a**) Localization uncertainty for a bent-strand defect. (**b**) Mixed detection results in a multi-defect scene. (**c**) Localization error for a spaced-strand defect accompanied by an additional false-positive prediction. (**d**) False-positive spaced-strand detections on normal cable texture.

**Table 1:** Experimental environment.

Configuration Item	Specification
Operating system	Linux 5.15.0-94-generic
CPU	Dual Intel Xeon Gold 6330 (2.00 GHz)
GPU	NVIDIA GeForce RTX 3090
Memory (GPU)	24 GB
Programming platform	Linux
Programming language	Python 3.10
Deep learning framework	PyTorch 2.2.2
CUDA	12.1
cuDNN	8.9.2

**Table 2:** Training configurations of representative detectors.

Model	Architecture/Variant	Input	Epochs/Selection	Batch	Optimizer	Initial LR	Weight Decay
YOLOv8n	Nano (n)	640	300	32	SGD	0.01	0.0005
YOLOv11n	Nano (n)	640	300	32	SGD	0.01	0.0005
RT-DETR	ResNet-18 (R18)	640	250	16	AdamW	1 × 10^−4^	1 × 10^−4^
DCGD-RTDETR	R18-based	640	250	16	AdamW	1 × 10^−4^	1 × 10^−4^
Faster R-CNN	ResNet-50-FPN	640	Best ckpt.: 25	4	SGD	0.005	0.0005

**Table 3:** Comparison of convolution operators.

Model				AP@0.5 (%)					mAP@0.5:0.95 (%)
	BS	WS	BT	LS	CR	SS	DP	mAP	
Original	90.7	98.4	76.5	80.4	97.7	89.1	95.4	89.7	56.2
DBB	88.3	98.4	78.8	84.7	97.3	90.9	97.6	90.9	56.1
FDConvC3	87.2	96.5	78.5	84.2	96.0	88.2	93.1	89.1	55.7
DualConv	87.4	95.8	84.2	84.4	96.2	88.5	98.4	90.7	56.6

**Table 4:** Comparison of downsampling modules.

Model				AP@0.5 (%)					mAP@0.5:0.95 (%)
	BS	WS	BT	LS	CR	SS	DP	mAP	
Original	90.7	98.4	76.5	80.4	97.7	89.1	95.4	89.7	56.2
SRFD	90.3	95.7	73.6	82.2	96.9	88.7	97.0	89.2	55.8
HWD	94.4	96.5	73.7	81.1	97.8	88.6	98.7	90.1	56.1
ContextGuidedDown	89.4	96.3	75.4	87.3	97.6	91.1	98.5	90.8	56.3

**Table 5:** Comparison of high-level feature regulation strategies.

Model				AP@0.5 (%)					mAP@0.5:0.95 (%)
	BS	WS	BT	LS	CR	SS	DP	mAP	
Original	90.7	98.4	76.5	80.4	97.7	89.1	95.4	89.7	56.2
Attention	90.7	96.5	75.9	80.6	96.4	89.1	99.1	89.8	55.7
AggregatedAtt	91.4	99.4	67.5	77.0	97.4	88.0	97.2	88.3	53.2
DyT	89.0	98.4	79.7	83.0	96.0	90.0	98.1	90.6	56.5

**Table 6:** Ablation study.

DualConv	CGDown	DyT	mAP@0.5 (%)	Parameters (M)	GFLOPs
			89.7	20.1	58.3
✓			90.7	16.1	48.6
	✓		90.8	22.5	63.1
		✓	90.6	20.0	58.3
✓	✓		91.7	18.9	53.9
✓		✓	91.1	15.9	47.3
	✓	✓	91.6	22.3	61.7
✓	✓	✓	92.4	18.6	53.4

Note: ✓ indicates that the corresponding module is included in the configuration.

**Table 7:** Comparison with representative object detectors.

Model				AP@0.5 (%)					AP@0.75/mAP@0.5:0.95 (%)
	BS	WS	BT	LS	CR	SS	DP	mAP	
YOLOv11n	87.4	98.7	65.5	64.7	89.6	87.6	95.5	84.1	50.7/49.2
YOLOv8n	73.0	96.5	65.1	64.2	84.7	78.1	94.5	79.4	47.9/47.8
Faster R-CNN	74.4	96.0	69.1	76.8	83.0	70.0	89.6	79.8	47.6/44.7
RT-DETR	90.7	98.4	76.5	80.4	97.7	89.1	95.4	89.7	61.7/56.2
DCGD-RTDETR	93.0	98.4	83.5	86.2	96.9	89.4	99.3	92.4	63.7/57.1

**Table 8:** Results on the RF100 Cable Damage dataset.

Model	mAP@0.5 (%)	mAP@0.5:0.95 (%)	Parameters (M)	GFLOPs
YOLOv11n	88.3	46.6	2.6	6.3
RT-DETR	90.6	45.4	19.9	56.9
DCGD-RTDETR	91.9	46.2	18.5	52.1

**Table 9:** Per-class error analysis of DCGD-RTDETR on the CableInspect-AD test set at a confidence threshold of 0.25 and an IoU matching threshold of 0.5.

Defect Class	GT	TP	FP	FN	Recall (%)
Broken strand	50	48	10	2	96.0
Welded strand	29	28	0	1	96.6
Bent strand	22	17	8	5	77.3
Long scratch	68	63	8	5	92.6
Crushed	40	38	7	2	95.0
Spaced strand	123	103	31	20	83.7
Deposit	61	61	1	0	100.0
Overall	393	358	65	35	91.1

**Table 10:** Statistical robustness across four paired random seeds.

Metric	Model	Seed 1	Seed 2	Seed 3	Seed 4	Mean ± SD	95% CI	*p*
mAP@0.5	RT-DETR	92.05	89.44	91.18	91.23	90.98 ± 1.10	89.23–92.72	0.086
	DCGD-RTDETR	92.09	91.56	92.27	92.27	92.05 ± 0.34	91.51–92.58	
AP@0.75	RT-DETR	66.85	63.37	64.79	65.04	65.01 ± 1.43	62.74–67.29	0.005
	DCGD-RTDETR	64.49	61.91	62.91	63.75	63.26 ± 1.11	61.50–65.03	
mAP@0.5:0.95	RT-DETR	59.28	56.33	58.94	57.41	57.99 ± 1.37	55.80–60.18	0.791
	DCGD-RTDETR	58.91	57.69	57.45	58.71	58.19 ± 0.73	57.03–59.35	

Note: All metric values are reported as percentages. SD denotes standard deviation and CI denotes the 95% confidence interval. *p*-values were obtained using two-sided paired *t*-tests across matched random seeds (1–4).

**Table 11:** Supplementary strict-IoU and inference-efficiency measurements.

Configuration	AP@0.75 (%)	Latency (ms/Image)	FPS	Timing Protocol
YOLOv11n	50.73	0.967	1034.33	Ultralytics inference-only
RT-DETR baseline	61.71	6.847	146.05	RT-DETR inference-only
DCGD-RTDETR	63.73	8.930	111.99	RT-DETR inference-only
DualConv + DyT	68.79	8.740	114.42	RT-DETR inference-only
CGDown + DyT	63.73	9.681	103.30	RT-DETR inference-only
Faster R-CNN	47.64	14.819	67.48	Batch 1; 50 warm-up images

Note: All timing measurements were obtained on an NVIDIA GeForce RTX 3090. The RT-DETR baseline, DCGD-RTDETR, DualConv + DyT, and CGDown + DyT values use inference-only timing from the RT-DETR evaluation pipeline. YOLOv11n uses the Ultralytics 8.3.9 inference timing, and Faster R-CNN was measured separately with batch size 1 after 50 warm-up images. Therefore, the YOLOv11n and Faster R-CNN values are included as practical references rather than strictly controlled cross-framework comparisons.

## Data Availability

The datasets analyzed in this study are publicly available from their original sources. The derived data and experimental results generated during the current study are not publicly deposited because they are part of an ongoing research project and are subject to project-related restrictions. These materials are available from the corresponding author upon reasonable request.
